# Sphynx

**Published:** 1875-02

**Authors:** 


### TO THE PUBLIC.

THE SPHYNX TOOTH PASTE COMPANY beg leave to state that they have finally solved the Problem which has for many years engaged the attention of the Dental Profession and of Chemists, viz.: The production of a Tooth Paste that should permanently retain its plastic and semi-fluid condition. Much time, money and thought have been directed towards this end, and also to the best method of putting up the Paste when manufactured. We have been so fortunate as to hit upon the best, and probably the only convenient form of doing this, by the adoption of the Metallic Tube, so well known to artists, and so admirably adapted to the preservation of their paints and colors in a moist state. The Tubes are filled by 40 lbs. pressure, and are air tight. The name of "SPHYNX" has been selected because of the durable and unchangeable properties of our preparation, which remains unaffected by any climate and by years of keeping. And also because of a permanent semi-fluid Tooth-Paste having so long remained a problem unsolved; even as the great Egyptian Sphynx has for thousands of years remained a mystery to the world's inhabitants, and the attempt to create such another miracle of art has never been made.

The market is full of preparations purporting to accomplish all sorts of impossibilities. We do not propose to obviate the necessity of the dentist, but merely to assist him in his professional duties.

This Preparation will not arrest the decay of the teeth (nor will any other). Judging from the labels of the leading articles in this line, the Dentist is of no use; we do not assert any nonsense of this kind, but merely present you with a preparation that will accomplish neither more nor less than we claim. It will cleanse and purify the teeth, flavor the breath, and prevent the accumulation of tartar. The convenient manner in which it is put up will be, we are sure, readily appreciated by all; it may be carried in the vest or dress pocket while on a journey. It will not break or spill contents if dropped, and will not get dry or lose fragrance, even though the Cap get lost. Another merit that distinguishes our preparation from all others, is that purchasers have the choice of flavors; prominent among them being Rose, Cleopatra, Frangipane, Millefleur, etc. The Caps are jeweled, and the goods made as attractive as possible, forming one of the chief ornaments of the druggists' counter. The large sized Tubes contain twice as much material as any ordinary box of tooth powder, and is the most economical preparation yet offered, there being absolutely no waste.

---

### DIRECTIONS.

Unscrew the Cap, and press the flattened end of the Tube between the thumb and finger, and catch the Paste upon a tooth brush *previously wet*. A half inch of the Paste as it issues from the tube will be sufficient, though as large a quantity as is desired may be used.

THE PRICE OF THE LARGE-SIZED TUBES IS FIFTY CENTS.

**For sale by all good Druggists.**

MANUFACTURED BY

**DRS. FORSTER & STOWELL, Patentees,**

1323 Arch Street, Philadelphia, Pa.

